# RAGE inhibitor TTP488 (Azeliragon) suppresses metastasis in triple-negative breast cancer

**DOI:** 10.1038/s41523-023-00564-9

**Published:** 2023-07-13

**Authors:** Melinda Magna, Gyong Ha Hwang, Alec McIntosh, Katherine Drews-Elger, Masaru Takabatake, Adam Ikeda, Barbara J. Mera, Taekyoung Kwak, Philip Miller, Marc E. Lippman, Barry I. Hudson

**Affiliations:** 1 Sheila and David Fuente Graduate Program in Cancer Biology, University of Miami Miller School of Medicine, Miami, FL USA; 2 Lombardi Comprehensive Cancer Center and Department of Oncology, Georgetown University Medical Center, Washington, DC USA; 3 Department of Cell Biology, Leonard M. Miller School of Medicine, University of Miami, Miami, FL 33136 USA

**Keywords:** Breast cancer, Metastasis, Target validation, Targeted therapies

## Abstract

Triple-negative breast cancer (TNBC) is a highly aggressive and metastatic cancer subtype, which is generally untreatable once it metastasizes. We hypothesized that interfering with the Receptor for Advanced Glycation End-products (RAGE) signaling with the small molecule RAGE inhibitors (TTP488/Azeliragon and FPS-ZM1) would impair TNBC metastasis and impair fundamental mechanisms underlying tumor progression and metastasis. Both TTP488 and FPS-ZM1 impaired spontaneous and experimental metastasis of TNBC models, with TTP488 reducing metastasis to a greater degree than FPS-ZM1. Transcriptomic analysis of primary xenograft tumor and metastatic tissue revealed high concordance in gene and protein changes with both drugs, with TTP488 showing greater potency against metastatic driver pathways. Phenotypic validation of transcriptomic analysis by functional cell assays revealed that RAGE inhibition impaired TNBC cell adhesion to multiple extracellular matrix proteins (including collagens, laminins, and fibronectin), migration, and invasion. Neither RAGE inhibitor impaired cellular viability, proliferation, or cell cycle in vitro. Proteomic analysis of serum from tumor-bearing mice revealed RAGE inhibition affected metastatic driver mechanisms, including multiple cytokines and growth factors. Further mechanistic studies by phospho-proteomic analysis of tumors revealed RAGE inhibition led to decreased signaling through critical BC metastatic driver mechanisms, including Pyk2, STAT3, and Akt. These results show that TTP488 impairs metastasis of TNBC and further clarifies the signaling and cellular mechanisms through which RAGE mediates metastasis. Importantly, as TTP488 displays a favorable safety profile in human studies, our study provides the rationale for evaluating TTP488 in clinical trials to treat or prevent metastatic TNBC.

## Introduction

Breast cancer is the most common malignant neoplasia and the second leading cause of cancer-related death in women^1,2^. Most breast cancer deaths are not due to the primary tumor but are caused by cancer cell metastasis to a secondary organ^3^. While triple-negative breast cancers (TNBC) account for only 10–15% of all breast cancers, they are the most aggressive, very heterogeneous, and have the lowest overall 5-year survival rate^4,5^. Adjuvant treatment options for TNBC are limited to chemotherapeutics as TNBCs lack expression of targets for endocrine and HER2-targeted therapies^5,6^. Unfortunately, chemotherapeutics have high toxicity due to off-target effects on normal tissues, and TNBC can become resistant to chemotherapy, leading to relapse and, ultimately, death from TNBC metastasis^7,8^. Recently, targeted therapies, including immune checkpoint and PARP inhibitors, have shown limited efficacy in TNBC; however, there are no effective long-term treatment options for metastatic TNBC^9–11^. Therefore, there is an urgent need for novel efficacious agents that target metastatic TNBC, display lower toxicity profiles, and improve the quality of life and survival of patients with TNBC.

The Receptor for Advanced Glycation End-products (RAGE) is critical for the progression and metastasis of various cancers^6,12–18^. RAGE is a pattern recognition receptor with many known ligands, including Advanced Glycation End-products (AGEs), numerous S100 family proteins (including S100A4, S100A8/9, S100A12, and S100B), HMGB1, Mac-1 (CD11b/CD18), and amyloid-β peptide^12,18–23^. RAGE expression is low in most cells and tissues in non-pathogenic states, with a prominent exception of lung type 1 alveolar epithelial cells^18,24^. However, upregulation of RAGE expression occurs in various inflammatory disease states where RAGE ligands accumulate, including diabetes, cardiovascular disease, neurodegenerative disorders (including Alzheimer’s disease), and various cancers^18,25–27^. Increased RAGE protein expression in human tumor tissue is associated with higher histological grade and poorer clinical outcomes in multiple cancers^28–31^. In breast cancer, higher RAGE protein levels are associated with increased risk of metastasis and lower survival^6,13,28,32^. Furthermore, human and murine TNBC cell lines display high RAGE expression, and RAGE knockdown inhibits cell migration and invasion in vitro^6,13,32^.

FPS-ZM1 is a RAGE-specific small molecule inhibitor that binds to and blocks ligand-mediated RAGE signaling^33^. Our prior work demonstrated that FPS-ZM1 reduced tumor growth and metastasis in a preclinical model of TNBC^13^ and impaired cell invasion in vitro without affecting cell viability^13^. The RAGE inhibitor TTP488 (Azileragon, PF-04494700), which also inhibits RAGE-ligand binding, is an orally bioavailable small molecule that, unlike FPS-ZM1, has undergone human clinical trials^34^. TTP488 has shown excellent safety profiles in clinical trials of Alzheimer’s disease, with initial Phase II data also showing that TTP488 may reduce cognitive impairment in patients with mild Alzheimer’s disease^35^. However, more extensive Phase III trials (NCT02080364, the STEADFAST Study) were discontinued due to the lack of efficacy of one of the trial’s primary end-points. Unfortunately a dose 4 times lower than a dose proven safe was employed and may explain the failure in the Phase III study^35^. The observation that deletion of the RAGE gene in mice is not lethal and does not affect development or fertility^36,37^ makes targeting RAGE in cancer plausibly safe, and its inhibitor TTP488, a highly translatable, potentially safe approach for anti-cancer therapy.

In the current study, we investigated if RAGE inhibition with TTP488 would inhibit the progression and metastasis of TNBC in vivo and in vitro, and we explored the underlying cellular and signaling mechanisms affected by RAGE inhibition. Using multiple in vitro and in vivo models of metastatic TNBC, we compared the effects of TTP488 and FPS-ZM1 on mechanisms of metastatic behavior and the development of metastasis in TNBC. Here, we show that TTP488 exerts a potent anti-metastatic effect greater than FPS-ZM1 in orthotopic xenograft and experimental metastasis models of TNBC. Transcriptomic analysis of primary tumor and metastatic lung tissues corroborated these findings, showing that RAGE inhibition impaired highly conserved driver mechanisms of the metastatic cascade. Moreover, validation of these mechanisms revealed that TTP488 directly inhibits tumor cell adhesion, invasion, and migration, without affecting cellular viability or proliferation. Further mechanistic analysis revealed that RAGE inhibition affects critical signaling pathways, growth factors, and cytokines intricately linked to metastasis. These results support the repursposed use, and rapid translation of TTP488 into clinical trials of metastatic TNBC.

## Results

### TTP488 impairs spontaneous metastasis in an orthotopic xenograft model

**Fig. 1:**
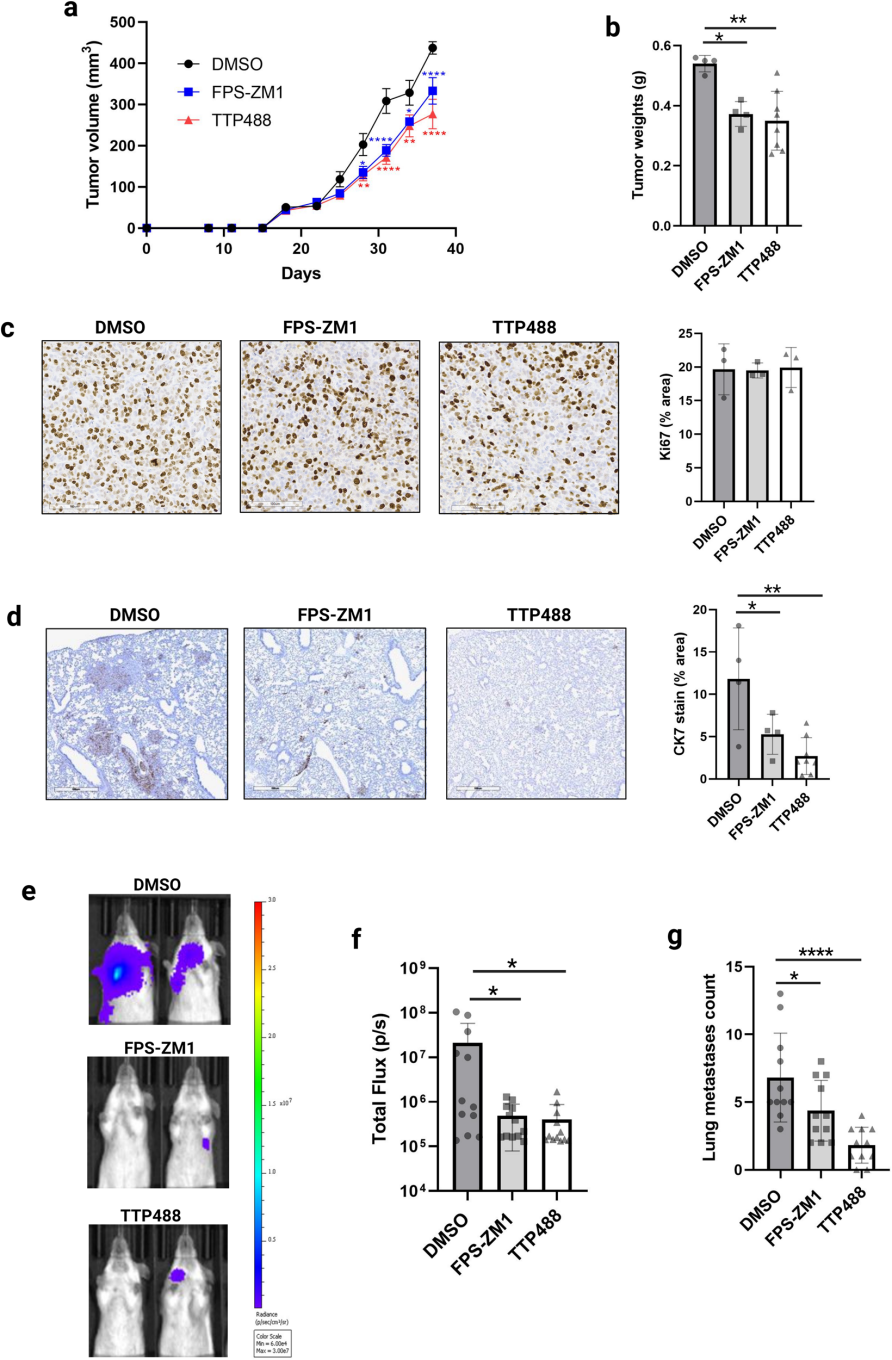
The effect of TTP488 and FPS-ZM1 on tumor growth and metastases in breast cancer mouse models. **a** 4175 cells were injected into the mammary fat pad of immunocompromised NSG mice, and mice were treated IP twice per week with 1 mg/kg TTP488 or FPS-ZM1 (or vehicle (DMSO) control). Tumor size was measured every 3 days, and (**b**) at the end of the experiment, tumors were weighed. Data shown are from 4–8 mice per group (repeated twice). **c** Immunohistochemical analysis of 4175/NSG tumors for proliferation (Ki67). **d** Immunohistochemical analysis of human CK7 to assess for metastasis in 4175/NSG mouse lung tissue. **e** 4T-1 cells were tail-vein injected into BALBc mice, and mice were treated IP twice per week with 1 mg/kg TTP488 or FPS-ZM1 (or vehicle (DMSO) control). Representative IVIS images at day 13 are shown from *n* = 11 per group. **f** Total flux measured from IVIS images is shown. **G** Surface lung metastases count performed at necropsy. Values shown are mean ± SD. Statistical analysis: one or two-way ANOVA with Dunnett’s multiple comparisons test. *p < 0.05; **p < 0.01; ***p < 0.001, ****p < 0.0001.

Our prior work demonstrated that both the human highly metastatic variant of MDA-MB231 (MDA-MB231-4175; herein 4175) and murine 4T1 TNBC cell lines have high RAGE expression^13^, and they both metastasize to the lungs from orthotopically implanted primary tumors^38,39^. We have also reported that genetic knockdown (shRNA) and pharmacological targeting (FPS-ZM1) of RAGE impaired TNBC metastatic behavior in vitro and in vivo^13^. Therefore, we chose these cell lines to compare the effects of TTP488 and FPS-ZM1 on metastatic TNBC cells. We have previously shown that FPS-ZM1 (1 mg/kg, twice weekly) significantly reduced lung metastasis in the 4175/NSG orthotopic model^13^. In the present study, we compared the efficacy of 1 mg/kg TTP488 to 1 mg/kg FPS-ZM1 in impairing tumor progression and metastasis. TTP488 and FPS-ZM1 had a similar modest but significant inhibitory effect on tumor progression in vivo, as reflected by tumor growth (Fig. 1a), with TTP488 (*p* = 0.002) affecting final tumor weight more potently than FPS-ZM1 (*p* = 0.01) (Fig. 1b). To assess mechanisms by which RAGE inhibition affects tumor growth, tumors were stained for Ki-67 to determine tumor proliferation (Fig. 1c). No antiproliferative effect of either drug was seen in vivo, as indicated by Ki-67 staining, compared with control-treated 4175/NSG tumor bearing mice.

We next analyzed the effect of RAGE inhibitors on tumor cell metastasis in lung tissue of 4175/NSG mice. Analysis of tumor cell metastasis by IHC of human CK7 (tumor-cell specific) in formalin-fixed lung sections demonstrated that 1 mg/kg TTP488 and 1 mg/kg FPS-ZM1 reduced lung metastasis, with TTP488 affecting metastasis more potently at this dose (*p* = 0.001 versus *p* = 0.04) (Fig. 1c, d).

### TTP488 impairs experimental lung metastasis in a 4T1/BALBc syngeneic tail vein injection model

Metastasis is a multi-step process comprising a sequence of events, from the primary tumor formation to the colonization of the metastatic site, known as the invasion-metastasis cascade^40^. To further dissect the role of RAGE in breast cancer metastasis, we used treatment of RAGE inhibitors in an experimental lung metastasis model in mice by lateral tail vein injection of TNBC cells^41^. To assess the effects of TTP488 and FPS-ZM1 on the colonization and growth in the lung by TNBC cells, we employed the syngeneic 4T1/BALBc model, as we have previously shown that RAGE gene knockdown affects metastasis from the orthotopic site using this model^13^.

Based on the drug efficacy in the 4175/NSG orthotopic model, we treated the mice with 1 mg/kg TTP488 or FPS-ZM1 twice weekly or DMSO as vehicle control. We performed IVIS imaging to visualize and quantitate changes in luminescence derived from the 4T1-Fluc cancer cells in the lung. Mice treated with either TTP488 or FPS-ZM1 had significantly reduced tumor burden (*p* = <0.05) in the lungs at day 13 compared to the vehicle control-treated mice (Fig. 1e, f). Surface lesions of the lungs counted at the time of necropsy supported the IVIS imaging results (Fig. 1g), with TTP488 (*p* = <0.0001) demonstrating a more significant effect on impairing metastasis than FPS-ZM1 (*p* = 0.04).

### Transcriptomic analysis of primary xenograft and metastatic tumors reveals that RAGE inhibition impairs multiple mechanisms driving TNBC metastasis

We next performed bulk RNA sequencing to assess the tumor-intrinsic mechanisms in the tumor and metastatic lung tissue to gain insight into the potential anti-metastatic mechanisms of TTP488 and FPS-ZM1. Using the tumor and metastatic lung tissue from 4175/NSG implanted mice allowed us to selectively assess ex vivo the tumor intrinsic (human) mechanisms affected by RAGE inhibition from host (mouse) effects.

**Fig. 2:**
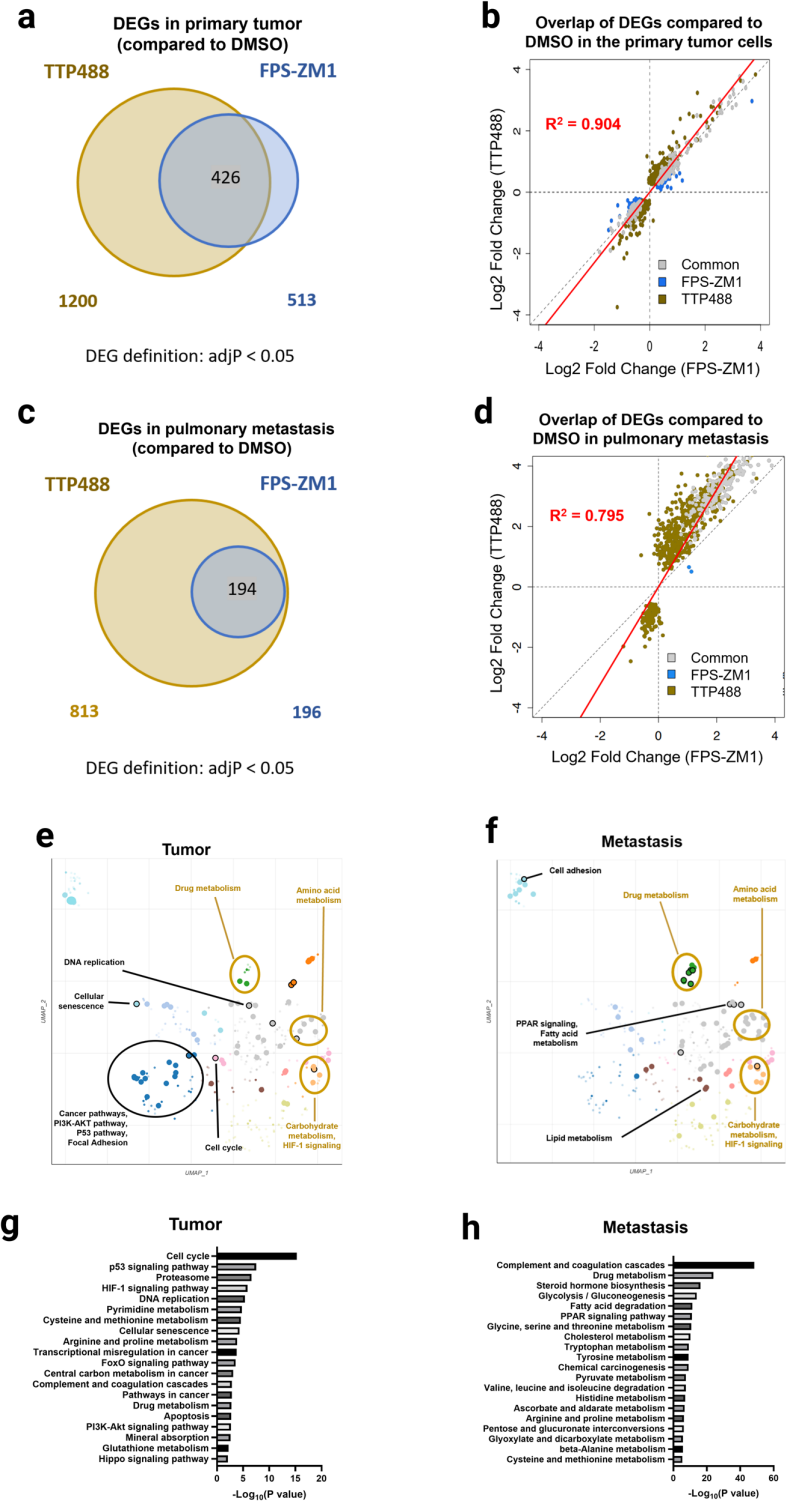
Transcriptomic and pathway analysis of the effects of TTP488 and FPS-ZM1 on primary tumor and metastasized tumor cells. **a** Venn-diagram of the overlap in Differentially Expressed Genes (DEGs) in the tumors of the two treatment groups (TTP488 and FPS-ZM1). **b** Scatterplot of the log2-fold change of DEGs calculated in one or both treatments relative to DMSO control in primary tumors. A trend line of plotted genes (red) juxtaposes a reference (dotted diagonal) line denoting equivocal expression fold change by both treatments. **c** Venn diagram of the overlap in DEGs in the TTP488 and FPS-ZM1 treated lungs. **d** Scatterplot of the log2 fold change of DEGs calculated in one or both treatments relative to DMSO control in metastatic lungs. **e**, **f** Scatterplot of the enriched terms in the KEGG 2021 Human library in tumor (**e**) and lung (**f**) for TTP488 treated mice versus vehicle control. Significantly enriched pathway terms overlapping between the primary tumor and the pulmonary metastasis are marked with gold circles and labels; non-overlapping significantly enriched terms are marked and labeled in black. **g**, **h** Bar graphs show the top 20 KEGG and GO BP cancer-associated enriched pathway terms in order from top to bottom by the magnitude of significance for genes significantly changed with TTP488 treatment relative to DMSO control in tumor versus lung metastasis.

In the primary tumor, we found 1200 Differentially Expressed Genes (DEGs) in the TTP488 group and 513 DEGs in the FPS-ZM1 treatment group compared to control tumors (DMSO treated) (Fig. 2a). In the TTP488 and FPS-ZM1 treatment groups, we identified 426 overlapping DEGs, with all gene changes occurring in the same direction (218 upregulated genes and 208 downregulated genes) (Fig. 4a). Comparison of the DEGs from the tumors of TTP488 and FPS-ZM1 treated mice, revealed complete concordance in gene changes and the direction of gene expression (Fig. 2b). Furthermore, when comparing the common genes significantly differentially expressed by both TTP488 and FPS-ZM1 based on Log2Foldchange, TTP488 appeared to alter gene expression more robustly (~1.2 fold) than FPS-ZM1 (R^2^ = 0.904) (Fig. 2b). In metastatic lung tissue compared to control, we found 813 DEGs in the TTP488 treatment group and 196 in the FPS-ZM1 treatment group, with 194 DEGs overlapping between TTP488 and FPS-ZM1 (Fig. 2c). As in primary tumors, both RAGE inhibitors displayed complete concordance in the direction of DEGs compared to DMSO control, with TTP488 treatment altering gene expression ~1.4 fold more robustly than FPS-ZM1 (R^2^ = 0.795) (Fig. 2d).

To explore the pathways through which RAGE inhibition limits tumor progression and metastasis, we performed functional enrichment analysis using KEGG pathway alignment on the significantly upregulated and downregulated DEGs from the TTP488-treated tumors (Fig. 2e, h, Supplementary Fig. 1). For pathway analysis, we focused on the TTP488 treatment group (versus control), as DEGs seen in the FPS-ZM1 treatment group were predominantly a subset of the DEGs in the TTP488 treatment group (Fig. 2a, c). Comparing all the DEGs in TTP488 treated primary tumors and their corresponding lung metastases-associated DEGs, we found that 17% of the tumor-associated DEGs overlapped with 26% of the metastasis-associated DEGs. KEGG enrichment analysis showed overlapping and non-overlapping pathways in the primary and the metastasized tumor cells. Hierarchical clustering of the enriched terms in primary tumor and pulmonary metastasis was visualized on scatterplots (Fig. 2e, f). Nine overlapping clusters emerged in both the tumor and the metastasis enrichment; Drug metabolism, Amino acid metabolism, Carbohydrate metabolism, and HIF-1 signaling-related pathway clusters were enriched in both the primary tumor and the metastasized tumor cells. Cell cycle, DNA replication, Pathways in cancer, PI3K-Akt signaling, p53 signaling were among the enriched pathways and clusters in the primary tumor that were not enriched in the metastastatic lesions due to TTP488 treatment (Fig. 2e). Conversely, in the metastasized cells Lipid metabolism, Fatty-acid metabolism, PPAR signaling, and Cell adhesion molecules were differentially significantly enriched (Fig. 2f). The top 20 cancer-associated significantly enriched KEGG pathways terms in the tumor and metastasis are shown in Fig. 2g, h. We further analyzed the top 100 significantly enriched pathways (FDR < 0.05), assessed from the GO Biological Processes, WikiPathway, KEGG, and MSigDB Hallmark Databases, as shown in Supplementary Fig. 1, which show similar pathway differences to those seen with KEGG. These differences between tumor and metastasis may indicate the differing mechanisms by which RAGE inhibition influences the primary tumor cells differently than the disseminated metastasized tumor cells.

### The effects of TTP488 and FPS-ZM1 on proliferation, viability, and cell cycle of TNBC cancer cells

**Fig. 3:**
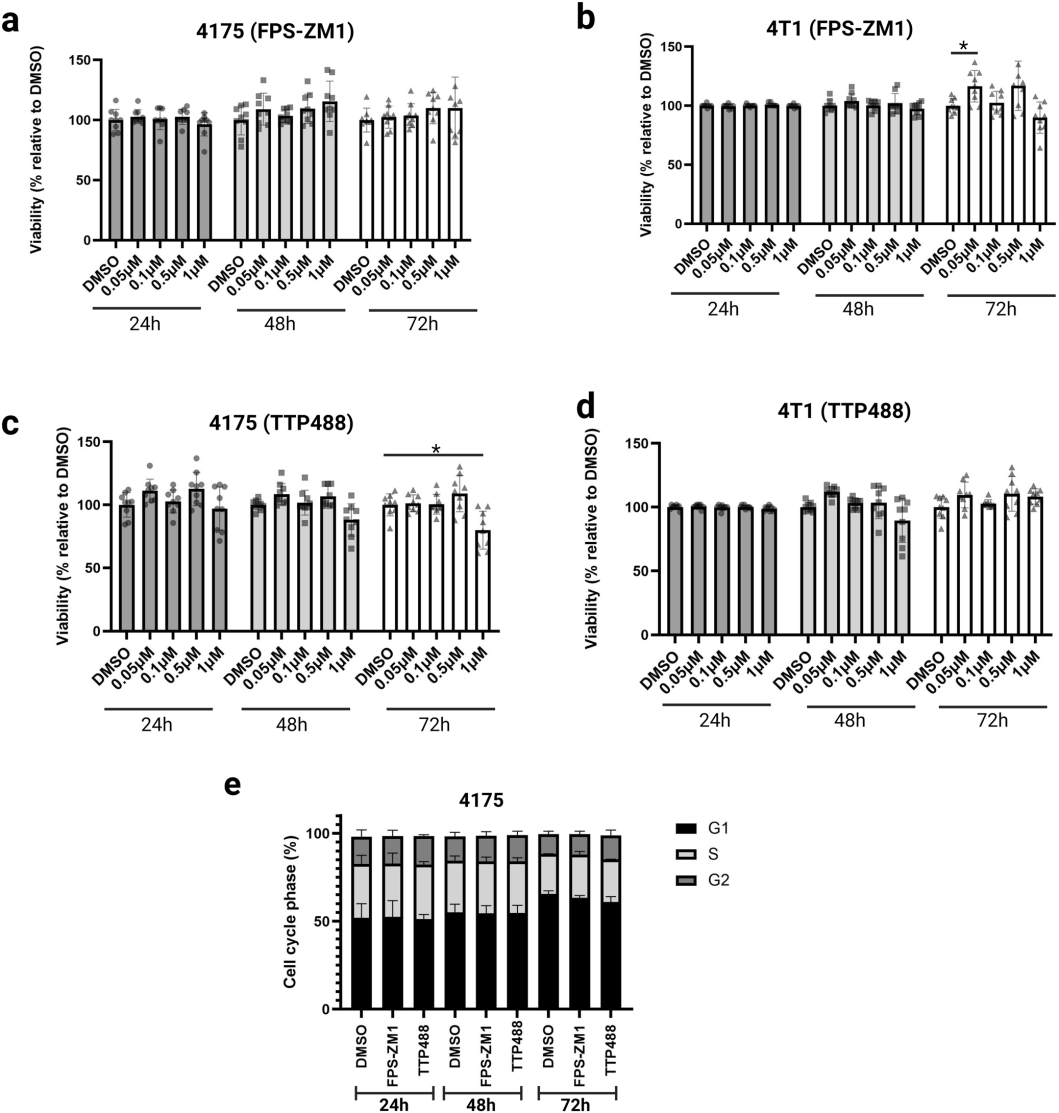
The effect of TTP488 and FPS-ZM1 treatment on tumor cell growth, viability, proliferation, and cell cycle in vitro. **a**–**d** Cell viability/proliferation assays were performed using the crystal violet assay. 4175 and 4T1 cells were treated with a range of concentrations (50–1000 nM) of FPS-ZM1 or TTP488 for 24, 48, or 72 h. **a** 4175 cell treatment with FPS-ZM1; (**b**) 4T1 treatment with FPS-ZM1; (**c**) 4175 treatment with TTP488; (**d**) 4T1 treatment with TTP488. **e** Cell cycle analysis of 4175 cells treated in vitro with 1000 nM of FPS-ZM1 or TTP488 for 24, 48, or 72 h. Cells were stained with propidium iodide solution and analyzed for cell cycle distribution by flow cytometry. Representative column graph showing the percentage of cells in each cell cycle. Values show mean + SD from 3–5 independent experiments performed in triplicate. Statistical analysis was performed with either one-way or two-way ANOVA. *p < 0.05.

RNAseq analysis of primary tumors indicated changes in cell growth, proliferation, and cell cycle. We, therefore next tested the effects of TTP488 and FPS-ZM1 on these cellular functions in vitro. To test the effects of RAGE inhibition on cell proliferation and viability, we conducted a dose-response study with a crystal violet assay that assesses cell viability in vitro. We used a range of drug concentrations between 50 nM and 1000 nM of FPS-ZM1 and TTP488 to treat 4175 and 4T1 cells for 24, 48, or 72 h (Fig. 3a–d). Neither TTP488 nor FPS-ZM1 affected cell proliferation or viability at any dose or time point (Fig. 3a–d). We next tested the impact of RAGE inhibitors on the cell cycle in 4175 cells. Cells were treated with either FPS-ZM1, TTP488, or vehicle control (DMSO) for 24, 48, or 72 h, and cell cycle phase distribution assessed by BrdU/propidium iodide staining and analysis by flow cytometry. We did not observe any effects of either RAGE inhibitor on cell cycle progression in vitro (Fig. 3e).

### TTP488 is a potent inhibitor of TNBC cell adhesion, invasion, and migration

**Fig. 4:**
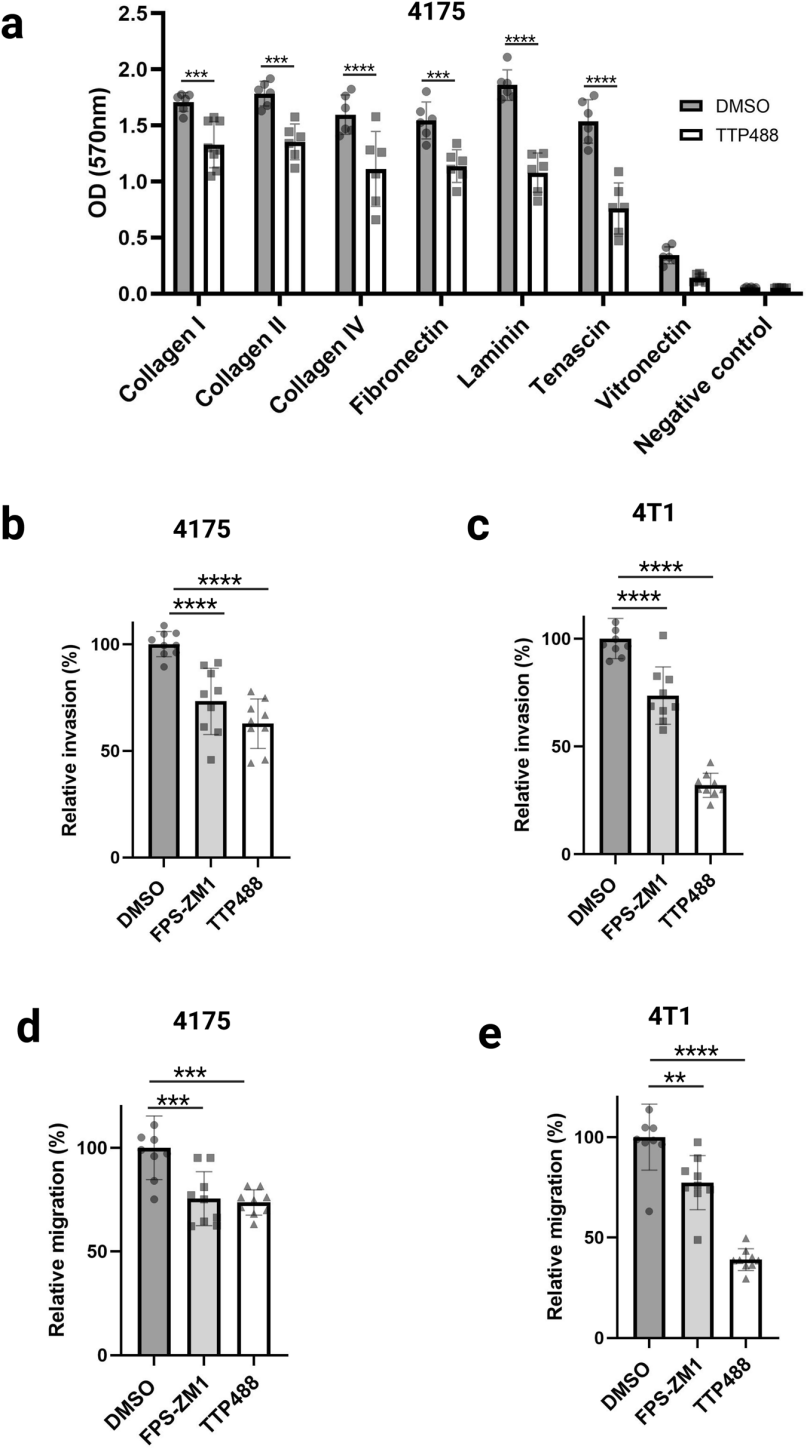
The effect of TTP488 and FPS-ZM1 treatment on tumor cell adhesion, migration, and invasion. **a** Cell adhesion to various extracellular matrix (ECM) component proteins was assessed using the ECM Cell Adhesion Array. 4175 cells were treated with TTP488 (1 µM) or vehicle control for 48 h, before adhesion assays. 4175 cells (150,000 cells per well) were incubated for 2 h at 37 °C in the ECM Array. Attached cells were stained with crystal violet and quantified. Data shows two experimental repeats run in triplicate. **b**, **c** Boyden chamber invasion assay of 4175 and 4T1 cells treated with FPS-ZM1 (1 µM) and TTP488 (1 µM). **d**, **e** Boyden chamber migration assay of 4175 and 4T1 cells treated with FPS-ZM1 (1 µM) and TTP488 (1 µM). Values show mean + SD from three independent experiments performed in triplicate. Statistical analysis was performed with either one-way or two-way ANOVA. **p < 0.01; ***p < 0.001, ****p < 0.0001.

Cell adhesion, migration, and invasion are essential attributes of cancer cells in the metastatic progression of solid tumors. Prior studies have demonstrated that RAGE signaling is critical for tumor cell adhesion, migration, and invasion^12,13,32,42^. As RNAseq pathway analysis of 4175/NSG tumors revealed changes in cell adhesion (Fig. 2e, f), we first tested the impact of RAGE inhibitors on these processes. To assess how RAGE inhibition affects cellular adhesion, we tested the impact of RAGE inhibition on the effect of tumor cell adhesion to multiple extracellular matrix (ECM) component proteins critical to tumor dissemination and metastasis. We used the ECM Cell Adhesion Array Kit, which includes ECM proteins central to the growth, progression and metastasis of breast cancer cells, including various collagens, laminins and fibronectin. 4175 cells were treated with either TTP488 or DMSO control for 48 h before adhesion assays were performed. RAGE inhibitor treatment of 4175 cells impaired cell adhesion to multiple ECM proteins, including collagens (I, II & IV), fibronectin, laminin, and tenascin (Fig. 4a). Although adhesion of 4175 cells to vitronectin was lower than other ECM proteins, no effect of TTP488 was seen on cell adhesion to vitronectin (Fig. 4a).

We next tested the impact of TTP488 and FPS-ZM1 on the invasion and migration of 4175 and 4T1 breast cancer cells. Based on our prior study^13^ and the above-presented results in the viability and toxicity assays, we treated the cells for 24 h with a concentration of TTP488 or FPS-ZM1 (1 µM) in transwell invasion/migration assays^13^. Both TTP488 and FPS-ZM1 treatments significantly decreased the invasion and migration of 4175 and 4T1 cells (Fig. 4b–e). Statistical analysis demonstrated that 1 µM TTP488 had a greater effect than 1 µM FPS-ZM1 in decreasing the invasion and migration of 4T1 cells in vitro (Fig. 1c, e).

### RAGE inhibitors TTP488 and FPS-ZM1 alter tumor intrinsic systemic effects critical for tumor progression and metastasis

**Fig. 5:**
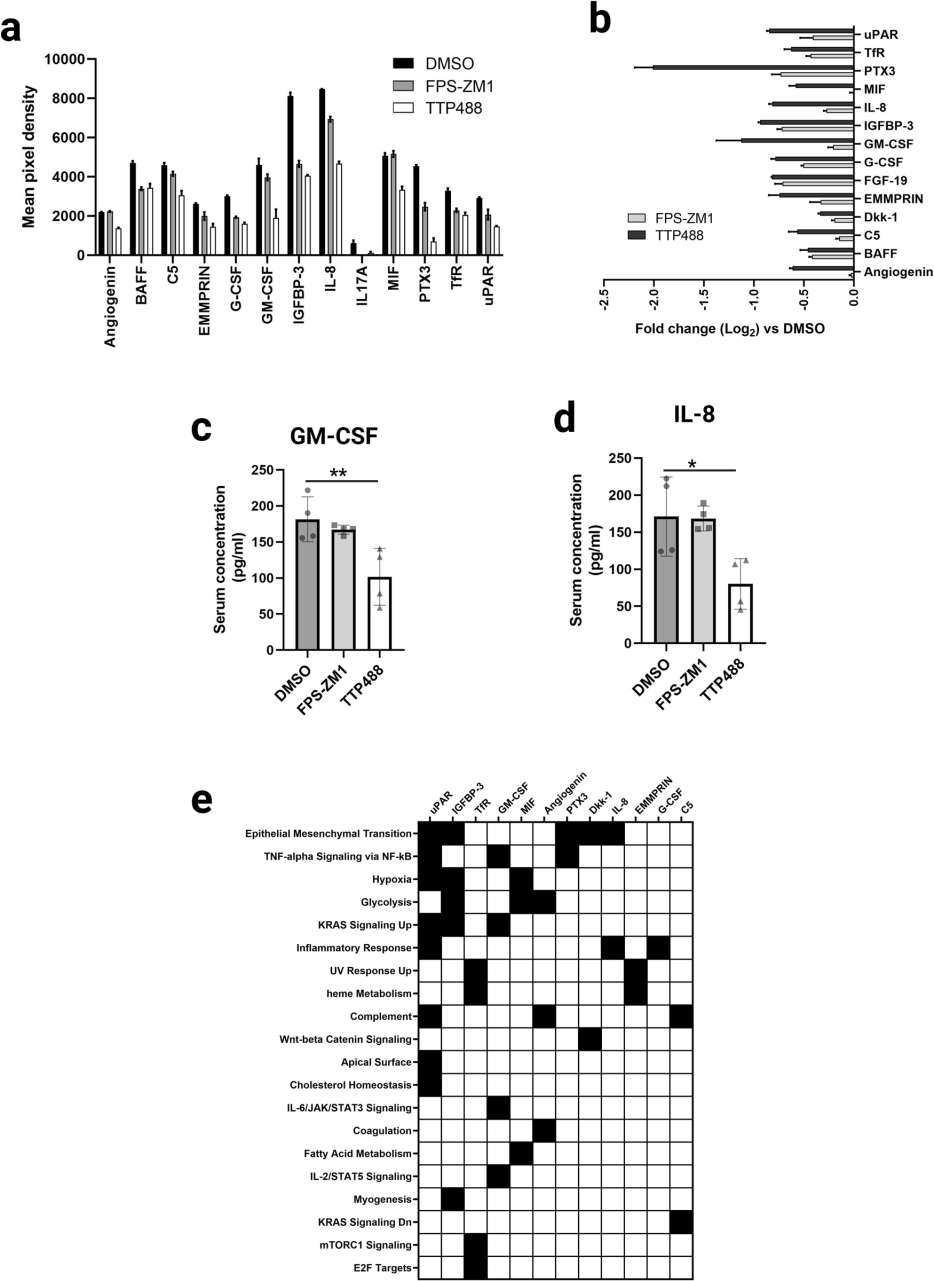
The effect of TTP488 and FPS-ZM1 on tumor-intrinsic changes in growth factor and cytokine levels in 4175/NSG tumor-bearing mouse serum. **a**, **b** Serum from 4175 tumor-bearing mice was assessed for changes in human tumor-intrinsic systemic changes due to RAGE inhibition by protein arrays. 3 pooled samples (for each of DMSO, FPS-ZM1 and TTP488) were assessed by the Proteome Profiler Human XL cytokine array; error bars represent two technical repeats per analyte. **a** All proteins that displayed differences compared to DMSO control by protein array (Proteome Profiler Human XL cytokine array) are shown. Data is shown as an average of mean pixel density for each pair of duplicate spots for each cytokine. **b** Log2-fold change in serum protein differences due to RAGE inhibitors relative to DMSO control. **c** GM-CSF ELISA was performed on serum from 4175 tumor-bearing mice. Samples (four per group) were run in duplicates for each individual sample. **d** IL8 ELISA was performed on serum from 4175 tumor-bearing mice. Samples (four per group) were run in duplicates for each individual sample. **e** Clustergram (of MsigDB Hallmark pathways) analysis generated by ENRICHR of tumor-derived protein changes in mouse serum.

We tested for various tumor-derived systemic changes in the serum from the 4175/NSG mice to investigate further the mechanisms by which RAGE inhibition may affect tumor cell metastasis. As the 4175 cells are of human origin, we used a human-specific array of cytokines, chemokines, and growth factors, allowing species-specific analysis of tumor cell-intrinsic mechanisms in the mouse. We showed that RAGE inhibitor treatment reduced the systemic levels of 14 tumor cell-derived factors in the treated mice compared to the DMSO control group (Fig. 5a, b). Both drugs inhibited 12 soluble factors, with only TTP488 affecting angiogenin and MIF levels (Fig. 5a, b). As seen by RNAseq, all RAGE inhibitor-induced changes were in the same direction for both drugs. Similarly to the RNAseq transcriptomic changes, TTP488 displayed a more potent inhibitory effect than FPS-ZM1 on protein expression (Fig. 5). ELISA of individual serum samples from 4175/NSG mice for GM-CSF (Fig. 5c) and IL8 (Fig. 5d), confirmed these data, showing TTP488 more potently affected serum levels of GM-CSF and IL8 compared to control DMSO mice. Pathway enrichment analysis based on the MSigDB Hallmark pathways^43^ showed that the 14 cytokines, chemokines, and growth factors contribute to many classical metastasis-promoting pathways, such as EMT, NFkB, JAK/STAT signaling and metabolic and inflammatory responses. Figure 5e clustergram shows the top 20 enriched tumor-intrinsic relevant pathways these cytokines modulate, including epithelial-mesenchymal transition (EMT), TNF-alpha signaling through NF-κB, hypoxia, and KRAS signaling (Fig. 5e).

### RAGE inhibitor TTP488 alters key signaling pathways critical for tumor progression and metastasis

RAGE-ligand binding can activate various intracellular signaling pathways, including mitogen-activated protein (MAP) kinases, JAK/STATs, and PI3K/Akt in various cells and tissues^18^. In cancer, RAGE intracellular signaling leads to changes in various pathways linked to adhesion, migration, invasion, proliferation, and cellular viability^18^. Our RNAseq data demonstrated that RAGE inhibition altered signaling through various pathways, including PI3K/Akt, HIF-1, and p53. Therefore, we performed an unbiased analysis at the phospho-protein level of the impact of RAGE inhibitors on tumor cell-intrinsic signaling with the human-specific Phospho-Kinase Antibody Array using tumors from 4175/NSG mice.

**Fig. 6:**
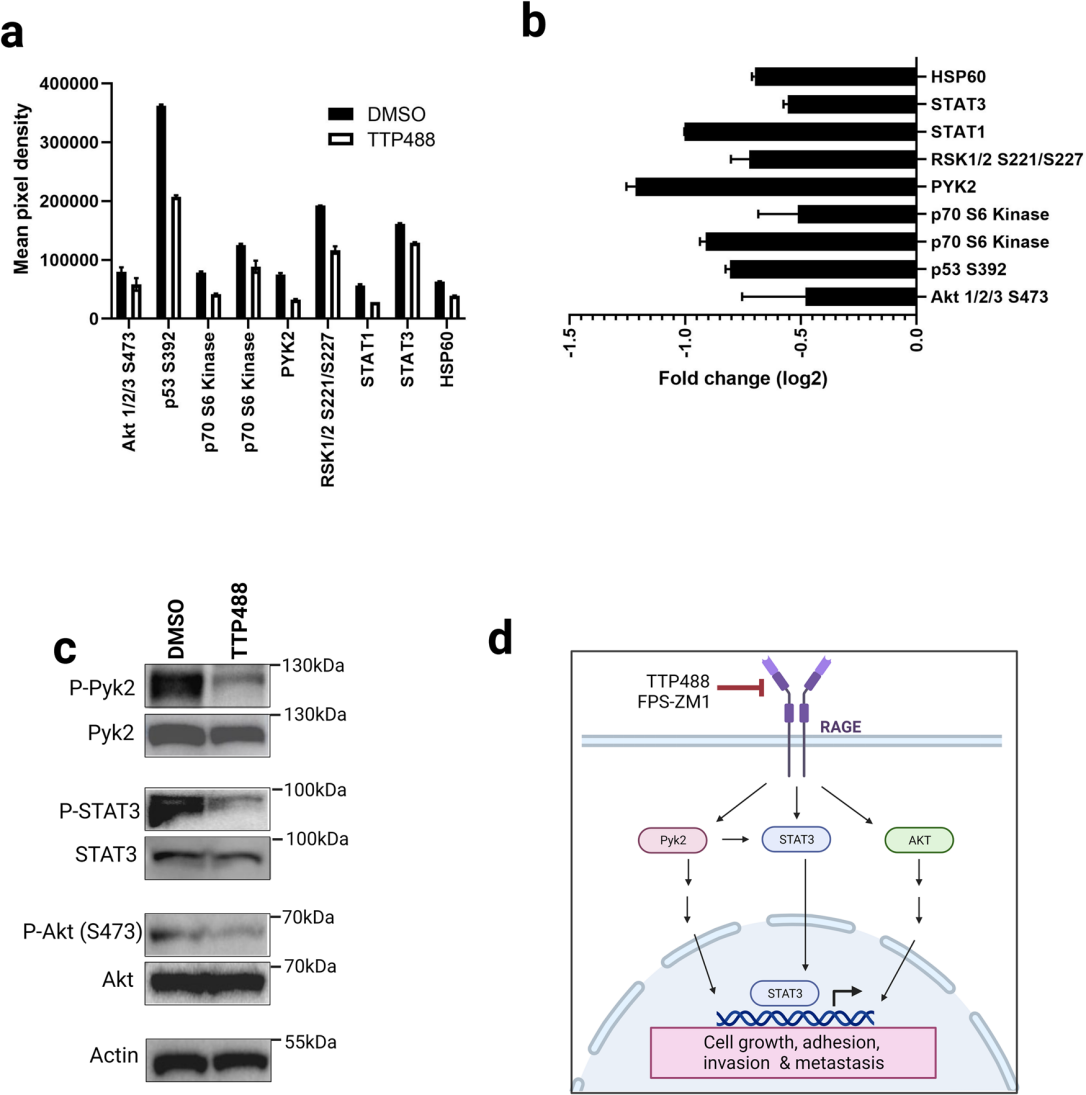
The effect of TTP488 and FPS-ZM1 on tumor-intrinsic signaling in 4175/NSG tumors. **a**, **b** Tumor lysate from 4175 tumor-bearing mice was assessed for changes in signaling pathway mechanisms due to RAGE inhibition by phospho-protein arrays. 3 pooled samples (for each of DMSO and TTP488) were assessed by the Proteome Profiler Human Phospho-Kinase Array Kit. **a** All phospho-proteins that displayed differences compared to DMSO control by protein array are shown. Data is shown as average of mean pixel density for each pair of duplicate spots for each cytokine. **b** Log2-fold change in phospho-protein differences due to RAGE inhibitor TTP488 relative to DMSO control. **c** Western blot validation of phospho-protein array changes was performed with tumor lysate from 4175 tumor-bearing mice (4 samples per condition). Representative images are shown for each condition. Samples were analyzed for phospho and total protein for Pyk2, STAT3 and AKT, and beta-actin loading control. **d** RAGE signaling in TNBC drives tumor metastasis. A schematic depicting the major signaling pathways activated by RAGE in TNBC cells leading to metastasis.

We showed that treatment with the RAGE inhibitor TTP488 reduced the activation of 9 phospho-protein levels in tumor lysate compared to the DMSO control group (Fig. 6a, b). These included various STAT proteins (STAT1 & 3), Pyk2, Akt, p53, p70 S6 kinase and RSK 1/2. Next, we validated several of these pathways by western blot for phospho/total levels with tumor lysate from 4175/NSG DMSO control versus TTP488 (4 samples per group). As shown in Fig. 6c, TTP488 suppressed the activation of Pyk2, STAT3, and Akt (representative images per group). We, therefore, propose from our transcriptomic and proteomic analysis that RAGE inhibitors block key tumor signaling pathways, including Pyk2, STAT3, and PI3K/Akt, leading to downreguation of gene expression of critical proteins critical for tumor cell growth, adhesion, migration, invasion and their subsequent metastasis to other organs (Fig. 6d).

## Discussion

In this study, we investigated whether the RAGE-specific inhibitor TTP488 impairs TNBC progression and metastasis compared to FPS-ZM1 and we explored mechanisms through which RAGE mediates breast cancer progression and metastasis. We show that both RAGE inhibitors reduced metastasis in multiple in vivo models, with TTP488 displaying a more potent inhibitory effect than FPS-ZM1 on metastasis at the same treatment dose. Transcriptomic analysis of tumor and metastatic tissue revealed that both RAGE inhibitors affected highly overlapping gene signatures and pathways, including cellular metabolism, cell cycle, cell adhesion, and apoptosis. Further, RAGE inhibition affected overlapping yet also distinct biology in the primary tumor cells versus the disseminated tumor cells at the transcript level. Additionally, we demonstrate that, in vitro, inhibition of RAGE with either TTP488 or FPS-ZM1 impairs metastatic driver mechanisms, including cell adhesion, migration, and invasion. Protein array analysis of tumor intrinsic secreted factors in serum revealed that RAGE inhibition downregulated proteins fundamental for driving metastatic processes, including angiogenesis, EMT, inflammation, and oxidative stress. Finally, phospho-proteomic analysis of 4175/NSG mice tumors revealed that RAGE inhibition affected critical signaling mechanisms that drive tumor progression and metastasis.

RAGE drives a range of proinflammatory states, including diabetes, cardiovascular disease, Alzheimer’s disease, and the progression and metastasis of various cancers^18,25–27^. RAGE is a highly desirable druggable target due to its heightened activity being restricted mainly to pathological states and the availability and widespread preclinical data on biologic and small molecule inhibitors^33,34,44^. Biological inhibitory agents against RAGE include the recombinant soluble ectodomain of RAGE (also known as sRAGE) and neutralizing monoclonal antibodies^12,44,45^. Indeed, sRAGE has been a critical tool in defining RAGE biology in vitro and in preclinical models of various human disease states^12,18,45^. For small molecule inhibitors of RAGE, the vast majority target the extracellular domain, which includes TTP488 (Azeliragon) and FPS-ZM1. TTP488 and FPS-ZM1 function by blocking RAGE-ligand binding and subsequent ligand-induced signal transduction^33,34^. TTP488 and FPS-ZM1 were developed independently by different groups for use and testing in preclinical models of Alzheimer’s disease^33,34^. Most importantly, TTP488 (developed by Transtech Pharma, now vTv Therapeutics) advanced to clinical trials and showed a favorable safety profile at 5 mg taken once daily in clinical trials of patients with Phase 2 mild Alzheimer’s disease^35^. Although Phase 3 trials were recently halted due to the lack of efficacy of one of the study’s primary end-points for Alzheimer’s disease, TTP488 is the only current RAGE-targeted therapy tested in human subjects. Therefore, TTP488 is a highly translatable therapy and may be immediately repurposable for clinical trials of other RAGE-driven pathological states, including cancer. Previously, we reported preliminary findings that FPS-ZM1 reduced tumor progression and metastatic burden in a xenograft model of TNBC^13^. Our previous study demonstrated that targeting tumor intrinsic RAGE function with either genetic approaches (shRNA gene knockdown) or inhibiting RAGE with FPS-ZM1 affects TNBC cell invasion without any detectable effects on cell viability or proliferation^13^. In the current study, we investigated the efficacy of the RAGE inhibitor TTP488 and performed in-depth testing and comparison to FPS-ZM1.

In the orthotopic xenograft 4175/NSG TNBC model, metastasis from the orthotopic site recapitulates the establishment of metastasis in patients, from the early stages of cancer cell dissemination to the later stages of overt metastasis. We found that TTP488 had a significant anti-metastatic effect, even greater than FPS-ZM1, at a dose of 1 mg/kg twice per week in the human xenograft 4175/NSG spontaneous breast cancer metastasis model. Interestingly, although RAGE inhibition with TTP488 and FPS-ZM1 significantly impaired lung metastasis, we observed a more moderate and comparable effect of TTP488 and FPS-ZM1 on primary tumor growth, as reflected by a modest reduction in tumor volume and final tumor weight. These data further support prior studies that RAGE plays a more significant role in breast cancer metastasis than tumor growth in vivo^6,13,16^. Therefore, to further explore the impact of RAGE inhibition on metastasis independent of primary tumor growth and tumor cell intravasation, we test TTP488 and FPS-ZM1 in an experimental tail vein injection model. Tail-vein inoculation of tumor cells causes them to become lodged in the lung’s small blood vessels and permits the study of extravasation and later stages of the metastatic cascade^46^. Using the well-established syngeneic metastatic 4T1/BALBc model^47^, we showed that both TTP488 and FPS-ZM1 significantly impaired metastasis in the tail-vein injection model. As seen with metastasis from the primary tumor site, TTP488 had a more potent inhibitory effect on metastasis than FPS-ZM1.

Prior data on the underlying tumor cell mechanisms by which RAGE drives metastasis has been limited to genetic models (RAGE overexpression and gene knockdown) and pathways of migration and invasion^12,13,48,49^. We, therefore, performed unbiased transcriptomic analysis on the primary tumors and metastatic lung tissues from NSG 4175 tumor-bearing mice treated with RAGE inhibitors. Consistent with the in vitro and in vivo phenotypes observed, TTP488 impacted the expression of more genes than FPS-ZM1. Most compelling, there was complete concordance between the expression and direction of the DEGs commonly altered by FPS-ZM1 and TTP488. This concordance speaks to the specificity of the drugs to RAGE and for blocking RAGE-ligands signaling, even though TTP488 and FPS-ZM1 are hypothesized to target different regions of the RAGE-ligand binding site and are structurally unrelated^18^.

In the primary tumor, we observed alterations in other major pathways involved in the metastatic cascade, including HIF1 signaling, cell cycle, and various pathways involved in cellular adhesion (adherens junction and focal adhesion). These data suggest RAGE inhibition in the primary tumor alters cell cycle, hypoxia response, PI3K/Akt and p53 signaling, and adhesion, in addition to migration and invasion of cancer cells^32,50,51^. While other studies have explored the mechanisms of RAGE signaling in the primary tumor, our study additionally analyzed mechanistic changes due to RAGE signaling in disseminated tumor cells (DTCs) in metastatic tissue. While we saw comparative effects of RAGE inhibition between DTCs and the primary tumor cells, which included cell adhesion, HIF-1 signaling/hypoxia, and various metabolic pathways, we did not detect differences in cell cycle, senescence, and various protumor signaling pathways (PI3K-Akt and p53). These data may reflect the differing biology required of a tumor cell to adapt to metastatic sites. During metastasis, only a small fraction of DTCs are able to colonize the secondary organ(s) and require distinct biological mechanisms than those required in the primary tumor^52^. These data, therefore, reveal that RAGE can target multiple anti-tumor mechanisms in both the primary tumor and later metastatic site.

In breast and other cancers, RAGE signaling can alter a broad range of tumor cell mechanisms, including increased cell adhesion, migration, invasion, proliferation, cell cycle changes, and resistance to apoptosis^12,16,48,49^. Interestingly, while we observed the effects of TTP488 or FPS-ZM1 on tumor growth in vivo, we did not observe any difference in cell growth assays in vitro. There are several possible explanations for this result. Firstly, it is possible that these results are due to inherent differences between the more homogenous in vitro analysis versus the more complex multicellular environment found in animal tumor models^53^. These data are consistent with prior studies of RAGE in TNBC, where in vivo tumor growth differences were seen but not reflected in in vitro cell studies of growth and proliferation^13^. Secondly, even the 3D in vitro culture of tumor cells can give different data from 2D culture. Prior studies have shown that 3D culture can be morphologically and molecularly closer to the in vivo state than 2D culture^53^. Our prior data on RAGE suggests that RAGE gene knockdown or inhibition in 3D soft agar assays can result in lower tumor cell growth^13^. Thirdly, our RNAseq data would support these in vivo differences in tumor cell growth, as RNAseq analysis revealed RAGE inhibitors impacted cell cycle, DNA replication, and cellular senescence in the primary tumor. However, ex vivo analysis of tumor proliferation by ki67 histology did not reveal any differences. Finally, 2D cancer culture typically lacks the various extracellular matrix proteins critical for tumor cell biology. To address these limitations, we performed other functional cell assays that may better reflect the complexity of the in vivo state. Critical to the dissemination and metastasis of tumor cells is their adhesion, migration, and invasion of the ECM of the tumor basement membrane and, ultimately, the metastatic tissue site. The breast tumor microenvironment comprises various cell types and a dynamic and complex ECM consisting of numerous proteins such as collagens, laminins, and fibronectin^54^. We found that RAGE inhibition impaired tumor cell adhesion to multiple ECM proteins critical to tumor progression and metastasis, including collagens, fibronectin, laminin, and tenascin. These ECM proteins are not only involved in the growth and progression of the primary tumor but also altered and present in the premetastatic niche to facilitate tumor cell metastasis^54^. While we did not assess direct interactions of RAGE with ECM proteins, prior work has demonstrated that RAGE can mediate cell movement by directly binding to various ECM proteins, including multiple collagens^42,55^, further potentially expanding the biological role of RAGE in tumor cell metastasis. In order for a tumor cell to metastasize from the primary site, the tumor cell must degrade and invade the vessel basement membrane matrix before extravasation and colonization of distant tissue sites. RAGE inhibition reduced transwell invasion assays through basement membrane Matrigel (composed of ECM proteins including collagens and laminin), and transwell chemotaxis assays for both 4175 and 4T1 cells. Together with our novel adhesion data, we have shown that RAGE inhibition affects multiple cellular stages of the metastatic cascade.

To delve further into the RAGE-mediated signaling mechanisms in driving tumor progression and metastasis, we performed various proteomic analyses of ex vivo tissue from the animal models. Given the differences between 2D culture models and in vivo models, we focused our studies on tissue and serum from animal models. We first assessed changes in tumor-secreted factors in the mouse serum and how RAGE inhibitors impacted anti-metastatic mechanisms. Interestingly, 12 different pro-metastatic proteins were identified to be downregulated with both TTP488 and FPS-ZM1. These proteins and their functions in breast cancer include cell migration and invasion (Dkk-1, GM-CSF, IL-8, PTX3), growth/proliferation (angiogenin, GM-CSF, G-CSF), stemness/EMT (BAFF, EMMPRIN) and survival (MIF);^42,56–61^ mechanisms all identified by our RNAseq and functional cellular studies. Further, many of these genes have been associated with poorer outcomes in breast cancer^42,56–61^.

We finally explored how RAGE inhibition regulates signaling mechanisms that underlie these pro-metastatic changes. RAGE is a major signal transduction receptor, shown in various cancers to function through various signaling pathways including MAP kinases, PI3K/AKT, and JAK/STATs. Further, most studies on RAGE signaling have been limited to the analysis of 2D in vitro tumor cell culture. Therefore, we performed an unbiased phospho-array analysis of tumors from 4175/NSG mice. The pathways identified to be altered by RAGE inhibition in the tumor included various STAT proteins (STAT1 & 3), Akt, p53, p70 S6 kinase, RSK 1/2, and Pyk2. These data overlap with transcriptional pathway changes identified by RNAseq in the tumor, including PI3K/AKT and p53. While most of these signaling molecules have been previously shown to be affected by RAGE, this is the first study to link RAGE signaling to Pyk2. Proline-rich tyrosine kinase 2 (Pyk2) is a non-receptor protein tyrosine kinase that regulates proliferation, migration, and invasion in numerous cell types^62^. Pyk2 is over-expressed in breast cancer, where it promotes tumor cell invadopodia formation and drives metastasis^62^. Further, it has been shown that Pyk2 can directly bind and activate STAT3^63^, also identified here to be altered by RAGE inhibition. We, therefore, propose that tumor cell RAGE signaling regulates Pyk2/STAT3 signaling, leading to alteration in tumor cell adhesion and invasion and, ultimately metastasis. Together, these protein and transcript studies reveal the complex interplay of RAGE in regulating signaling and cellular mechanisms of the metastatic cascade.

In the current study, we focused on tumor intrinsic driven mechanisms, however, there are limitations with these approaches. Prior studies have demonstrated that, despite the clear tumor intrinsic role of RAGE, RAGE is also expressed on non-tumor cells that participate in tumor progression and metastasis. This is not surprising since RAGE is upregulated in immune and endothelial cells under pathological states, and RAGE has also been shown to affect tumor malignancy through non-cancer cells of the tumor and metastatic microenvironment. RAGE signaling promotes tumor progression and metastasis by recruiting proinflammatory and immunosuppressive myeloid immune cells in the microenvironment through augmenting angiogenesis, and modulating cross-talk with cancer cells and non-cancer cells^13,51,64,65^. Indeed, our in vivo studies with RAGE inhibitors likely represent the dual-targeting of both cancer and non-cancer cells, despite the lack of critical immune cells in the NSG immunocompromised model. Our in vivo analysis of tumor-derived cytokines gives insight into this mechanism, despite the NSG mouse model lacking critical immune cells involved in tumor metastasis. While many of the secreted proteins regulated by RAGE affect tumor-intrinsic mechanisms that promote metastasis, they also modulate the tumor microenvironment and metastasis through modulating host mechanisms. These include factors that affect angiogenesis (angiogenin)^66^ and immune cell differentiation in the bone marrow and their recruitment to the tumor and metastatic site (G-CSF, GM-CSF, IL-8, MIF)^57,58,61,67^. Future studies are therefore required to dissect further the role of RAGE in cancer cell-intrinsic versus non-cancer cell-related mechanisms in the metastatic cascade. Due to RAGE playing a role in metastasis through distinct mechanisms in both the tumor and non-tumor cells, this further emphasizes RAGE as an attractive target against metastatic TNBC through the ability to target multiple factors involved in metastasis.

In conclusion, we show for the first time that TTP488 has anti-metastatic effects in multiple preclinical breast cancer models. We demonstrate novel signaling and functional mechanisms regulated by RAGE signaling and therefore identify new RAGE-mediated targets in breast cancer metastasis. Therefore, these data provide the first evidence for a new application and the rationale for rapid translation of the small molecule RAGE inhibitor TTP488 (Azeliragon) for use in metastatic breast and other cancers.

## Methods

### Compounds

RAGE-specific small molecule inhibitors FPS-ZM1 (4-Chloro-N-cyclohexyl-N-(phenylmethyl)benzamide; molecular weight 327.8) was purchased from MilliporeSigma (cat# 553030) and TTP488 (Azeliragon) (3-[4-[2-butyl-1-[4-(4-chlorophenoxy)phenyl]imidazol-4-yl]phenoxy]-N,N-diethylpropan-1-amine), molecular weight (532.12) was purchased from MedChemExpress (cat# HY-50682/CS-5117). Both compounds were dissolved in cell culture grade dimethyl sulfoxide (DMSO, cat# D2438, Sigma-Aldrich) and diluted with PBS to working concentration for animal studies, or with the appropriate cell culture media for in vitro studies.

### Cell culture

MDA-MB-231/4175 cells (herein referred to as 4175 cells) are a highly lung metastatic derivative of MDA-MB-231 (a human triple-negative breast cancer cell line), as previously described^39^. 4175 cells were obtained from Andy Minn as previously described^39^. 4T1 murine breast cancer cells (a triple-negative breast cancer model) were purchased from ATCC (cat # CRL-2539)^47^. 4175 cells were cultured in DMEM (cat# SH30243.01, Cytiva) supplemented with 10% Foundation Fetal Bovine Serum (FBS, cat# 900-108, Gemini). 4T1 cells were cultured in RPMI 1640 (cat# 10-040-CV, Corning) supplemented with 10% FBS in a humidified incubator with 5% CO_2_ at 37 °C. 4T1 cells were stably infected with firefly-expressing lentiviral particles (cat# LVP283, Amsbio) and selected with neomycin, to enable their in vivo tracking in experimental metastasis assays. To distinguish from the original 4T1 cells, these luciferase-expressing cells are named throughout the manuscript as 4T1-Fluc. 4T1-Fluc cells were only used in experimental metastasis assays in mice.

Cell cultures were routinely tested for mycoplasma by using MycoAlert sample kit (cat# LT37–618, Lonza). Additionally, mycoplasma negative status and cell line authentication by STR profiling were performed by IDEXX BioAnalytics.

### Animal studies

All animal studies were approved by the Institutional Animal Care and Use Committee of the University of Miami or Georgetown University. NSG (NOD.Cg-Prkdcscid Il2rgtm1WjI/SzJ; NSG) and BALB/cJ mice were purchased from Jackson Laboratory (Bar Harbor, ME, USA). Power analysis for all animal experiments was performed using Statmate 2.0 (Graph Pad software). All major animal experiments were repeated. For each animal experiment, different investigators were involved as follows: a first investigator (GHH) performed tumor cell implantations, a second investigator (MM) performed drug treatments of mice, a third investigator (BM or animal facility technician; unaware of treatment conditions) performed tumor measurements of mice or IVIS.

#### Orthotopic xenograft model

8 × 10^5^ human 4175 cells were injected in 100 μl sterile PBS with 4% Matrigel (cat# 354230, Corning) into the fourth inguinal mammary fat pad of 8-week-old female NSG mice. Sample sizes for mouse studies were 4–8 per group for xenograft studies in NSG mice. These numbers are based on tumor growth and metastasis differences with RAGE gene knockdown and inhibition in our prior study^13^. Animals were assigned random numbers and randomized for treatment after tumor cell implantation. The mice were treated with FPS-ZM1 or TTP488 (1 mg/kg) or DMSO (1%, vehicle control) intraperitoneally (I.P.) twice per week. Primary tumor growth was measured with calipers every three to five days, and tumor volume was calculated using the formula: V = length × width^2^ × 0.5. Mice were euthanized when tumor reached ~500 mm^3^ or when showing signs of health issues or distress. Euthanasia was performed with a lethal dose of ketamine-xylene followed by cervical dislocation. The primary outcome was tumor growth with metastasis assessed ex vivo as a secondary outcome.

#### Experimental metastasis model

2.5 × 10^4^ 4T1-Fluc cells were injected in 100 µL of PBS into the lateral tail vein of 8-week-old female BALB/cJ mice. One day before cell inoculation, mice were prophylactically treated once with 1 mg/kg of FPS-ZM1 or TTP488, or 1% DMSO in PBS I.P. After tumor cell implantation, mice were randomized to groups (*n* = 11 per group) and treated with either 1 mg/kg of FPS-ZM1 or TTP488, or 1% DMSO in PBS was administered twice per week. The primary outcome was the change in lung metastasis as a result of RAGE inhibition. Pulmonary metastatic progression was followed and quantified with bioluminescence imaging using an IVIS Spectrum in vivo imaging system (PerkinElmer). Mice were anesthetized with isoflurane before IVIS imaging at day 13 post tumor cell injection. Mice were euthanized under anesthesia by cervical dislocation. Quantification of bioluminescent images was performed by comparing total flux of the regions of interests (ROIs) using Living Image software (PerkinElmer).

### Histopathology and immunohistochemistry

For evaluation of the 4175 xenograft tumors and metastases, primary tumors and lungs from mice were harvested following euthanasia, fixed in 10% formalin, and embedded in paraffin for histopathological analysis. To quantitate the rate of pulmonary metastasis, lung sections were stained with hematoxylin and eosin (H&E) and immunohistochemistry against human cytokeratin 7 (cat# PA0942, Leica Biosystems, Buffalo Grove, IL, USA) by Georgetown HTSR core facility. Stained slides were imaged with an Aperio automated slide scanner, and images were analyzed (including % metastasis) with Qupath v0.3 analysis software.

### Bulk RNA sequencing and processing of RNA expression data

RNA extraction and paired-end RNA sequencing were performed by GENEWIZ using an Illumina HiSeq4000 platform from 4175/NSG primary tumor samples treated with DMSO, TTP488, or FPS-ZM1. Two biological replicates from each treatment group were sequenced. Sequence reads were trimmed to remove possible adapter sequences and nucleotides with poor quality using Trimmomatic v.0.36. Trimmed reads were mapped to the *Homo sapiens* GRCh38 reference genome, available on ENSEMBL, using the STAR aligner v.2.5.2b. Unique gene hit counts were calculated by using the featureCounts function from the Subread package v.1.5.2. Data normalization and differential gene expression (DEG) analysis were performed in R using the DESeq2 package. Using the Benjamini-Hochberg method for multiple testing adjustment, DEGs were identified as genes with an adjusted *p*-value of *P* < 0.05. Pathway enrichment analysis was performed on DEGs and stratified by the direction of significant change, using EnrichR (https://maayanlab.cloud/Enrichr/)^68^. The Kyoto Encyclopedia of Genes and Genomes (KEGG) database was used to determine the enrichment of the DEGs in canonical signaling pathways^69^. Bar graphs of the top 10 enriched pathways were created with Enrichr’s Appyter function^68^.

### Cell growth and viability assays

#### Crystal violet assay

Cell lines were plated for crystal violet cell growth and viability assay (1.5 × 10^4^ cells/well) in 24 well cell culture plates, as previously described^13^. Cells were plated for 24 h before compounds were added to the cells in serial dilution (0.05–1 μM; versus 0.1% DMSO vehicle control) in culture medium. Compounds at each dilution were evaluated in triplicate; cells treated with DMSO (0.1% final concentration) served as the vehicle control. Following the incubation period (24, 48, 72 h) with the compounds, the cells were fixed with 4% paraformaldehyde and stained with 0.1% crystal violet (Sigma) for 20 min. Crystal violet stain was extracted using 10% acetic acid, and cell growth was quantified in each by absorbance measurement at 595 nm (FLUOstar Omega microplate reader, BMG LABTECH). The measured absorbance was plotted as a relative % change compared to DMSO vehicle control.

#### Cell cycle analysis

To analyze cell cycle distribution, bromodeoxyuridine (BrdU) – propidium iodide (PI) staining was performed. Briefly, cells were plated at 7.5 × 10^4^ cells per 6-well plate (7800/cm^2^), and allowed to attach overnight. Once attached, media was replaced and cells were then treated with vehicle control (DMSO) or RAGE inhibitors at 1 µM for 48 h. During the final hour of culture, cells were pulsed with 10 µM BrdU (BioLegend Catalog# 423401), collected by trypsinization, fixed in iced-cold 70% ethanol, and stored at −20C for at least 2 h. Fixed cells were labeled with anti-BrdU-FITC (BioLegend Catalog# 364104) following manufacturer’s instructions, and counterstained with propidium iodide (p4170-25MG Sigma). Labeled cells were analyzed by flow cytometry (BD Fortessa), and results analyzed using FlowJo software.

### Cell adhesion array

Cell adhesion assays were performed using the ECM Cell Adhesion Array kit (cat# ECM540, EMD Millipore) according the manufacturer’s instructions. 4175 cells were pretreated with 1 µM RAGE inhibitors (TTP488) or 0.1% DMSO for 48 h in culture media. Cells were detached from tissue culture plates using Accutase cell detachment solution (cat# AT104, Innovative Cell Technologies, Inc.), and 1.5 × 10^5^ cells/well seeded in triplicate to each well of the ECM Array Plate. Cells were then incubated for 2 h at 37 °C in a CO2 incubator. Cells were fixed in Cell Stain Solution (EMD Millipore) for 5 min, and the stain was extracted using Extraction Buffer (EMD Millipore), and total cell adhesion per well quantified in each by absorbance measurement at 595 nm (FLUOstar Omega microplate reader, BMG LABTECH). The measured absorbance was plotted as OD compared to both DMSO vehicle control, and a negative control well containing BSA.

### Cell invasion and migration assays

Cell invasion assays were performed using transwell migration chambers, as described previously^13^. Briefly, 4175 and 4T1 cells were pretreated with 1 µM RAGE inhibitors (FPS-ZM1 or TTP488) or 0.1% DMSO for 1 h in culture media. 4175 cells (1.5 × 10^4^ cells) and 4T1 cells (6.5 × 10^4^ cells) were seeded in the upper chamber of 8-μm porous transwell inserts (cat# 662638 ThinCerts; Greiner bio-one) coated with 12.5 μg of Growth Factor Reduced Matrigel matrix (cat# 354230, Corning), in serum-free DMEM or RPMI, and incubated in 24-well plates with 1% FBS as a chemoattractant for 24 h. RAGE inhibitors or DMSO control were added to the appropriate complete media in both the upper and lower chambers. Following incubation for 16 h, cells were fixed with methanol for 10 min and stained with 0.1% crystal violet in dH_2_O. Non-invaded cells and excess stain were rinsed with water and removed from the inner surface of the insert with a cotton swab. To quantify the invaded cells, the insert membranes were imaged at 2x magnification (SteREO Discovery microscope, Zeiss), and the invaded area % was quantified with ImageJ 1.34n software (National Institutes of Health, USA). To assess cell migration, the same transwell assay method was used without Matrigel coating of the transwell inserts, as we have previously reported^42,70^.

### Cytokine array

Mouse serum cytokine and chemokine levels were analyzed with Proteome Profiler Human XL Cytokine Array kit (cat# ARY022B R&D systems) following ’manufacturers’ standard protocol. 50 µl of serum were pooled from three mice per group and applied to each membrane. Array images were analyzed and quantitated with GeneTools software (Syngene). Using the gene names of the identified proteins, pathway enrichment analysis and clustering was performed based on the MSigDB hallmark gene set using EnrichR software. The presented clustergram was created based on and identical to the EnrichR result using GraphPad Prism v9 software (Graphpad software) showing the protein names.

### Phospho-protein array

Tumor phospho-protein expression was analyzed with Proteome Profiler Human Phospho-Kinase Array kit (cat# ARY003C, R&D systems) following ’manufacturers’ standard protocol. Protein lysates from three primary tumor samples per treatment group of 4175/NSG tumors were prepared as per manufacturer’s protocol in Lysis Buffer 6. Protein concentration of each lysate were measured with BCA Kit (cat# 23227, Pierce). 100 ug of tumor lysate was pooled from three individual mouse tumors per group (DMSO versus TTP488) and applied to each membrane. Array images were analyzed and quantitated with GeneTools software (Syngene).

### Western blot

Tumor lysate was generated from 4175/NSG tumors as above for 4 individual mouse tumors per group (DMSO versus TTP488). Western blotting was performed using Invitrogen NuPAGE system as previously described^13^. Representative images are shown from each group. Primary antibodies (and dilutions) used were as follows: phospho-Pyk2 (Y402), 1:1000, (Cell Signaling; 3291), total-Pyk2, 1:1000, (Cell Signaling; 3480), phospho-STAT3 (Y705), 1:2000, (Cell Signaling; 9145), total-STAT3, 1:1000, (Cell Signaling; 9139), phospho-Akt, 1:1000, (S473) (Cell Signaling; 4060), total-Akt, 1:1000, (Cell Signaling; 9272), β-actin, 1:4000, (Cell Signaling; 3700). Secondary antibodies used were as follows: Goat Anti-Rabbit IgG H&L (HRP), 1:10000, (Abcam; ab97080), Goat Anti-Mouse IgG - H&L (HRP), 1:10000, (Abcam; ab97040). Western blots were visualized using Pierce ECL Western Blotting Substrate (cat# 32106, ThermoFisher) and a G:Box mini (Syngene).

### Statistical analysis

All data analysis and visualization were performed using GraphPad Prism v9 software (Graphpad software). For experiments comparing more than two groups or conditions, a one-way or two-way ANOVA test was used, followed by Dunnett’s multiple comparison test. For experiments comparing two groups or conditions, a two-tailed Student’s *t*-test was used. No animals or data points were excluded from analysis. Normality of experimental data was assessed by Graphpad Prism. All data are expressed as ±SD. A *p*-value of *p* ≤ 0.05 was considered statistically significant. Significance values: ****P < 0.0001; ***P < 0.0005; **P < 0.005; *P ≤ 0.05. All figures were created with BioRender.com.

### Reporting summary

Further information on research design is available in the Nature Research Reporting Summary linked to this article.

## Supplementary information

Supplementary Information

Reporting Summary

## Data Availability

All data associated with this study are presented in the article or supplemental materials. All RNAseq data files were deposited in the Gene Expression Omnibus (GEO) (accession number GSE214268; reviewer access token = yrghqiuqbzwlfon).
