# Supplementary material for: RAGE inhibitor TTP488 (Azeliragon) suppresses metastasis in triple-negative breast cancer
Source: NPJ Breast Cancer. 2023 Jul 13;9:59. doi: 10.1038/s41523-023-00564-9 (PMC10344964; doi:10.1038/s41523-023-00564-9)

Supplementary Figures

Supplementary Figure 1

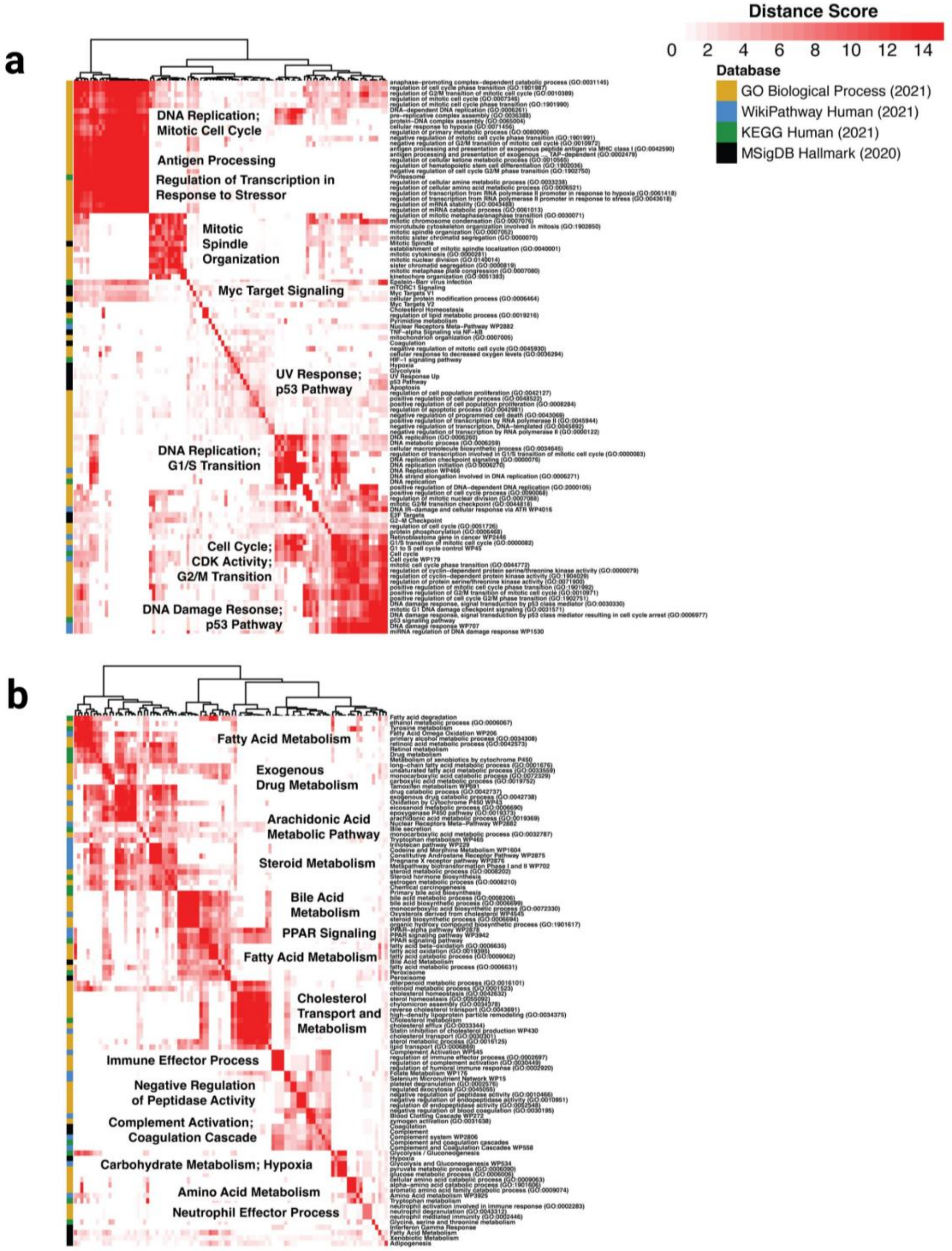

Supplementary Figure 1. Pathway enrichment analysis consisting of all genes differentially expressed in TTP488-treated (A) primary orthotopic tumors or (B) lung metastasis relative to DMSO control. The top 100 significantly enriched pathways (FDR<0.05), assessed from the GO Biological Processes, WikiPathway, KEGG, and MSigDB Hallmark Databases, were clustered via a distance metric derived from shared significant genes common amongst enriched gene sets. The origin of each of the top 100 enriched pathways are noted on the left of the plot. A manual summarization of highly clustered pathway processes is provided for readability.

Supplementary Methods

A broader approach of pathway enrichment analysis was performed by using EnrichR[1]. In R[2]. All differentially expressed genes (FDR < 0.05) were used in the analysis. In addition to the KEGG database[0], annotated gene sets from the GO Biological Process database[3,4], the WikiPathways database[5], and the MSigDB Hallmark database[6] were assessed for gene enrichment. The R package, *GeneSetCluster*[7], was used to calculated the distance between enriched gene sets based on common differentially expressed genes contained within each gene set and that contribute to its individual enrichment significance. The top 100 gene sets, enriched in TTP488-treated orthotopic primary tumors and lung metastasis versus DMSO control, respectively, were plotted by unsupervised clustering using the R package, *pheatmap*. Clustered enriched pathways were summarized manually.

Supplementary References

1) Kanehisa, M. and Goto, S.; KEGG: Kyoto Encyclopedia of Genes and Genomes. Nucleic Acids Res. 28, 27-30 (2000).
2) Kuleshov, M.V., *et al.* Enrichr: a comprehensive gene set enrichment analysis web server 2016 update. *Nucleic Acids Res* **44**, W90-97 (2016)
3) R Core Team (2022). R: A language and environment for statistical computing. R Foundation for Statistical Computing, Vienna, Austria. URL https://www.R-project.org/.
4) Gene Ontology Consortium. The Gene Ontology resource: enriching a GOld mine. Nucleic Acids Res. 2021 Jan 8;49(D1):D325-D334. doi: 10.1093/nar/gkaa1113.
5) Ashburner M, Ball CA, Blake JA, Botstein D, Butler H, Cherry JM, Davis AP, Dolinski K, Dwight SS, Eppig JT, Harris MA, Hill DP, Issel-Tarver L, Kasarskis A, Lewis S, Matese JC, Richardson JE, Ringwald M, Rubin GM, Sherlock G. Gene ontology: tool for the unification of biology. The Gene Ontology Consortium. Nat Genet. 2000 May;25(1):25-9. doi: 10.1038/75556.
6) Pico AR, Kelder T, van Iersel MP, Hanspers K, Conklin BR, Evelo C. WikiPathways: pathway editing for the people. PLoS Biol. 2008 Jul 22;6(7):e184. doi: 10.1371/journal.pbio.0060184.
7) Liberzon A, Birger C, Thorvaldsdóttir H, Ghandi M, Mesirov JP, Tamayo P. The Molecular Signatures Database (MSigDB) hallmark gene set collection. Cell Syst. 2015 Dec 23;1(6):417-425. doi: 10.1016/j.cels.2015.12.004.
8) Ewing, E., Planell-Picola, N., Jagodic, M. *et al.* GeneSetCluster: a tool for summarizing and integrating gene-set analysis results. *BMC Bioinformatics* **21**, 443 (2020).

Supplementary Figure 2

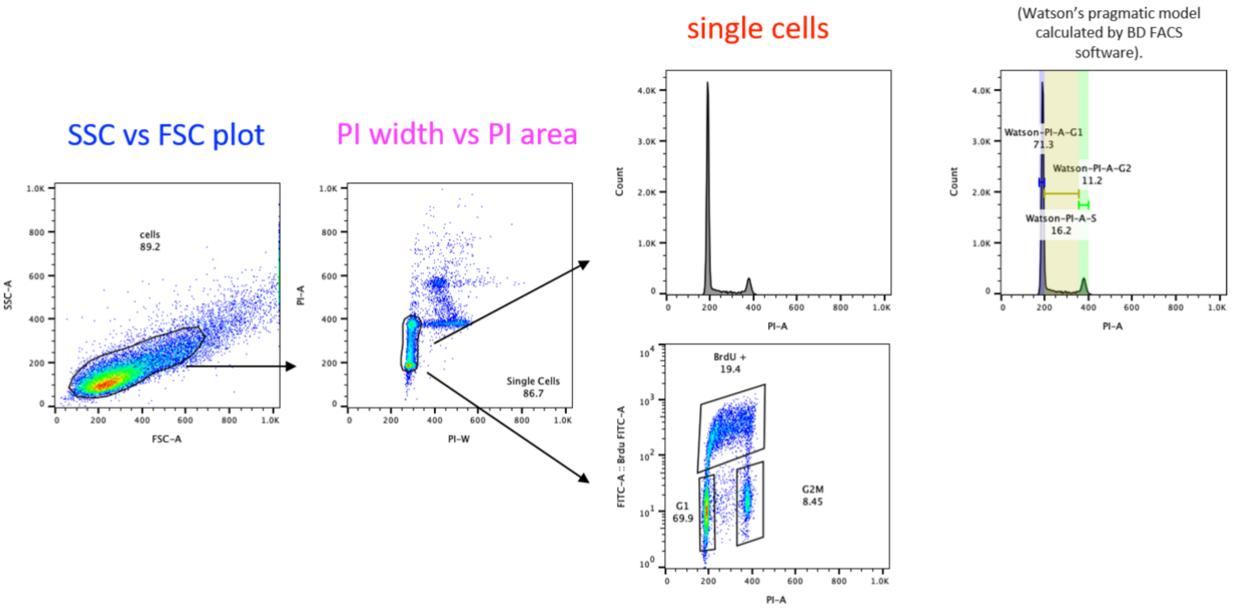

Supplementary Figure 2. Gating strategy used in cell cycle analysis of 4175 cells by flow cytometry. A standard gating strategy was used for the flow cytometric analysis of cell cycle. Debris was excluded on SSC vs FSC plot, and doublets were discriminated by plotting PI width vs PI area. 2N DNA content was arbitrarily set at 200 on the PI-area axis. Cell cycle profile of viable single cells was plotted as a histogram (PI-area), and cell cycle phase distribution was analyzed. Similarly, incorporation of BrdU (FITC) in viable single cells was plotted vs DNA content (PI- area) to simultaneously assess cell cycle distribution and BrdU incorporation (BrdU-FITC area vs PI area) on this population.

Source Files

Figure 6C

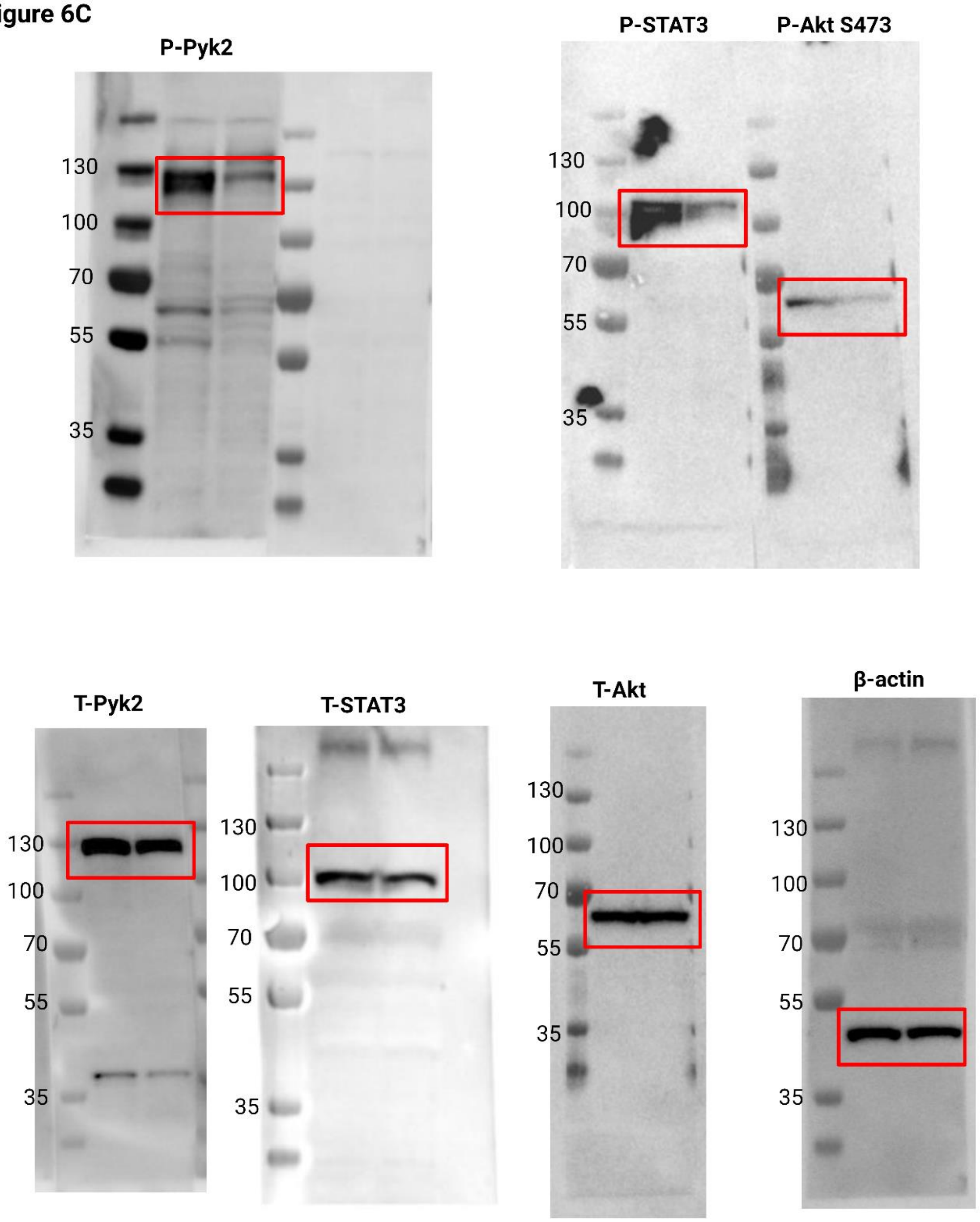

Supplement: Supplementary file 1 — Supplementary Information [file 41523_2023_564_MOESM1_ESM.pdf]
